# Contacting authors to retrieve individual patient data: study protocol for a randomized controlled trial

**DOI:** 10.1186/s13063-016-1238-z

**Published:** 2016-03-15

**Authors:** Areti Angeliki Veroniki, Sharon E. Straus, Huda Ashoor, Lesley A. Stewart, Mike Clarke, Andrea C. Tricco

**Affiliations:** Li Ka Shing Knowledge Institute of St. Michael’s Hospital, 209 Victoria Street, East Building, Toronto, ON M5B 1T8 Canada; Department of Geriatric Medicine, University of Toronto, Toronto, ON Canada; Centre for Reviews and Dissemination, University of York, York, UK; Northern Ireland Hub for Trials Methodology Research, Queen’s University Belfast, Belfast, UK; Epidemiology Division, Dalla Lana School of Public Health, University of Toronto, Toronto, ON Canada

**Keywords:** Meta-analysis, Patient-level data, Individual patient data, Individual participant data, Incentive, Data retrieval, Data collection, Response rate

## Abstract

**Background:**

Individual patient data (IPD) meta-analysis is considered the “gold standard” for exploring the effectiveness of interventions in different subgroups of patients. However, obtaining IPD is time-consuming and contact with the researchers responsible for the original trials is usually required. To date, there are no studies evaluating different strategies to optimize the process for retrieval of IPD from such researchers. Our aim is to examine the impact of providing incentives to the researchers responsible for the trials eligible for a meta-analysis to submit their IPD.

**Methods/Design:**

We updated our previously published systematic reviews for type 1 diabetes mellitus comparing long- and intermediate-acting insulin regimens (from January 2013 to June 2015) and for Alzheimer’s dementia comparing cognitive enhancers (from January 2015 to May 2015). Eligible were randomized controlled trials (RCTs) fulfilling the eligibility criteria of the systematic reviews. We will randomly allocate authors of the reports of these RCTs into an intervention or control group. Those allocated to the intervention group will be contacted by email, mail, and phone, and will be asked to provide the IPD from their RCT and will be given a financial incentive. Those allocated to the control group will be contacted by email, mail, and phone, but will not receive a financial incentive. Our primary outcome will be the proportion of authors who provide the IPD. The secondary outcomes will be the time to return the dataset (defined as the period between the information request and the authors’ response with the dataset), and completeness of data. We will compare the response rates in the two groups using the odds ratio and the corresponding 95 % confidence interval. We will also use binary logistic regression and cox regression analyses to examine whether different RCT characteristics, such as study size and sponsor information, influence the probability of providing IPD and the time needed to share the data.

**Discussion:**

This study will determine whether a financial incentive affects response rates when seeking IPD from the original researchers. We will disseminate our findings in an open access scientific journal and present results at national and international conferences.

**Trial registration:**

This trial is registered in Clinical Trials.gov, ID number NCT02569411. Date of registration 5 October 2015.

## Background

Over the past 30 years, there has been a considerable increase in the number of published systematic reviews and meta-analyses [1, 2], and systematic reviews have become the base unit for developing clinical practice guidelines as well as other knowledge tools used in clinical practice and policy-making. Conducting a meta-analysis requires accessing relevant outcome measurements from the individual, eligible studies. However, often outcome data from eligible studies are not available, as many authors do not report them in their manuscript.

Medical journals have attempted to deal with this difficulty by endorsing standards for reporting of study results, such as the Consolidated Standards of Reporting Trials (CONSORT) checklist [3]. Despite these efforts, it has been shown that study data are inconsistently reported; missing evidence is a substantial problem and one of the greatest threats to the validity of results from a systematic review and meta-analysis [4–6]. Authors of systematic reviews, therefore, have to contact authors of the original studies to attempt to obtain the relevant data for their analysis. However, authors of the original studies may be unwilling or unable to share their data [7] and hence, systematic review authors may have to exclude these studies from the meta-analysis. This inability to obtain data occurs frequently, and it might occur for a variety of reasons. For example, the original study authors may worry that a re-analysis might show an error or a pattern they missed [8], they may have limited publication rights for the data as a study sponsor owns them [9], they may have moved to a different university and lost the data, or the data might be old and saved in an inaccessible storage device [10].

Meta-analysis can be conducted using individual patient data (IPD) (data from each individual participant enrolled in each included trial) and/or aggregated data (summary point estimates from all participants enrolled in each included trial). Meta-analysis of IPD is considered the “gold standard” approach [11] for meta-analysis, in part because it provides the opportunity to explore differences in treatment effects across different subgroups. These subgroups might include subsets of patients, such as males and females, or subsets of studies, such as those conducted in different geographical locations [12]. Knowledge about the effectiveness of interventions in different subgroups is particularly important for decision- making. To date, there has been an increase in the frequency of published IPD meta-analyses [13], but authors of these studies (or data managers) may have to devote a lot of time and effort to obtain and prepare the IPD in the required format [14]. For instance, a previously conducted IPD meta-analysis of 19 studies included four research coordinators (who invested between 5 and 20 % of their full time) required 2088 hours of data management and more than 1000 emails between research coordinators and the data managers [15]. A main barrier to undertaking an IPD meta-analysis is that study authors usually report aggregated data, and IPD can only be obtained by contacting the original study authors for information that they did not include in their reports. Although there is a strong movement to share anonymized IPD from randomized controlled trials (RCTs) [16–19], this has not yet been well-established and the cooperation of the original study authors is crucial for providing the data in a usable format and answering queries about their data. For example, a recent planned IPD meta-analysis failed to be conducted, as the majority of the primary study authors did not share their data [20]. Given that obtaining IPD is important but also time-consuming, efforts need to be undertaken to understand how to optimize this process. Although previous studies have shown that financial incentives may improve response rates in survey requests [21–24], to the best of our knowledge there are no studies evaluating whether a financial incentive may facilitate the retrieval of IPD from authors of studies that are eligible for a systematic review.

The objective of our study is to examine the impact of providing a financial incentive to authors of RCTs that are eligible for a systematic review and meta-analysis, versus usual contact strategies to obtain the IPD. This trial protocol is in accordance with the Standard Protocol Items: Recommendations for Interventional Trials (SPIRIT) 2013 statement [25] and is registered in ClinicalTrials.gov with NCT02569411 (5 October 2015).

## Methods

### Study design

Our study will be a pragmatic (or effectiveness) RCT comparing the financial incentive (i.e., intervention) against the standard process of contacting authors to obtain IPD (i.e., control). The participants will be the authors of RCTs included in our two systematic reviews for type 1 diabetes mellitus [26] and for Alzheimer’s dementia [27], and we will randomize these authors in two groups to request their IPD (see Control and intervention groups section).

We updated the literature search for our published systematic reviews for type 1 diabetes mellitus [26] from January 2013 to June 2015, and for Alzheimer’s dementia [27] from January 2015 to May 2015. Briefly, we used the terms from our previous reviews to search MEDLINE, Cochrane Central Register of Controlled Trials, and EMBASE. Gray literature (i.e., difficult to locate and unpublished studies) was searched via trial registry websites, relevant society/association websites and conference abstracts. Reference lists of included studies and relevant reviews were also scanned. We used the Synthesi.SR tool [28] to screen citations and full-text articles. To ensure reliability, we conducted a training exercise before screening titles and abstracts using our eligibility criteria. When high agreement (greater than 90 %) was observed, two team members screened each title and abstract for inclusion, independently (level 1). After pilot-testing, the same reviewers independently screened the full text of potentially relevant articles to determine inclusion (level 2). Conflicts were resolved by team discussion.

In the type 1 diabetes study, we included trials studying adults (aged 18 years or older) with type 1 diabetes and comparing long-acting basal insulin analogue preparations with other long- or intermediate-acting insulin. We included RCTs of any duration reporting glycosylated hemoglobin and severe hypoglycemia outcomes. Our updated search identified 179 citations with 15 potentially eligible studies, whereas 4 RCTs met the inclusion criteria. In total, 30 RCTs were included in the updated type 1 diabetes study, where 30 studies evaluated glycosylated hemoglobin and 24 assessed severe hypoglycemia.

In the Alzheimer’s dementia systematic review, we included adults (aged 18 years or older) with Alzheimer’s dementia diagnosed using various criteria (e.g., Diagnostic and Statistical Manual of Mental Disorders, Nursing Minimum Data Set criteria). Again, we focused on RCTs of any duration and we included the Mini-mental State Examination and overall serious adverse events outcomes. Our updated search identified 73 citations with 12 potentially eligible studies, whereas 1 RCT met the inclusion criteria. Overall, 108 RCTs were eligible for the updated Alzheimer’s dementia review, where 74 studies provided data on the Mini-mental State Examination outcome and 64 provided data on the serious adverse events outcome.

### Participant recruitment

Corresponding authors of RCTs included in our previous and updated systematic reviews will be eligible for inclusion. We will attempt to obtain IPD from all eligible studies by contacting the corresponding author of each included RCT. In cases where the identified studies do not report authors’ email addresses or include non-working email addresses, we will search authors’ publications, PubMed, and profiles that are publicly available, including Research Gate and Google Scholar, to find contact information.

**Fig. 1:**
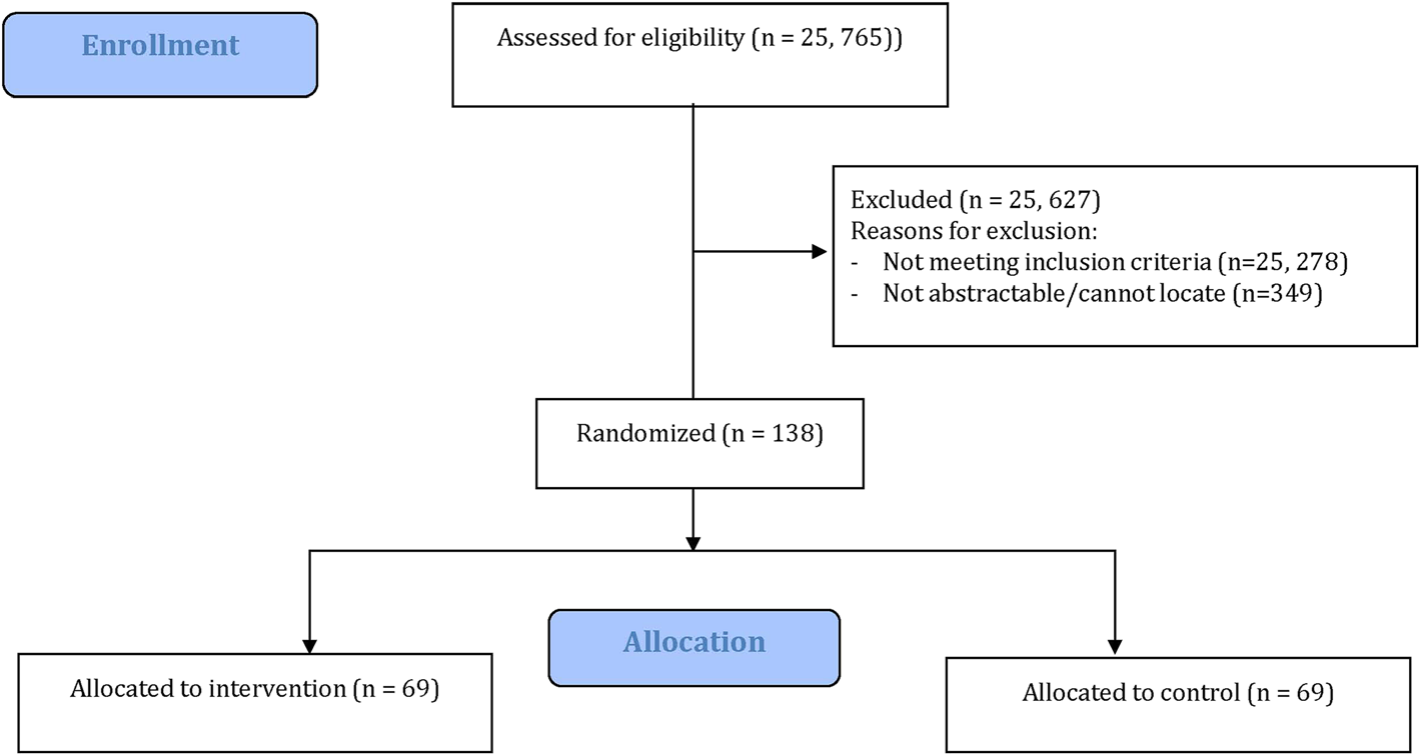
Consolidated Standards of Reporting Trials (CONSORT) flow diagram of the process of the randomized controlled trial

A challenge of this approach is that each author can only be contacted to ask for IPD from a single study. If a corresponding author of an eligible study has published more than one study, we will contact the first, last or the next in order author as presented in the paper. If a single author is included in more than one paper, then we will only contact him/her once for the newest study and the older study will be excluded. In such a case, for the IPD review and at the end of the RCT, we will contact the authors in the same way to obtain the IPD for all excluded studies. All authors who provide feedback during the conduct of the updated systematic review and IPD network meta-analysis for the type 1 diabetes study and the Alzheimer’s dementia study, will become part of an active collaboration and will be included in the authorship in the final publication (only if they agree) [29, 30]. This is in accordance with the International Committee of Medical Journal Editors (ICMJE) criteria [31]. A CONSORT flow diagram depicting the process of the study is presented in Fig. 1 [3]. This flow chart will be updated showing the flow of participants in the entire trial in the main manuscript.

### Randomization and blinding

Eligible authors will be randomized to one of the two trial groups using a 1:1 procedure. Randomization will be performed using a computer-generated random number list, and adequate allocation concealment will be ensured as the sequence will not be revealed until the end of the study. The computer randomization will be done centrally and conducted by a statistician (AAV) who will be blinded to the authors’ names. However, it is impossible to blind the corresponding authors and research personnel who will be in contact with them due to the nature of intervention. Blinding of outcome assessors is also not possible in this design.

### Control and intervention groups

#### Control group

**Fig. 2:**
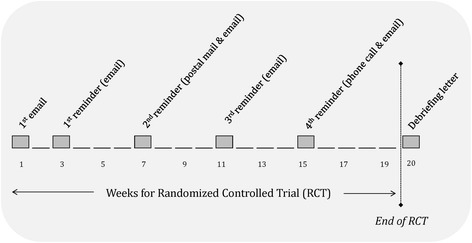
Study process flow diagram

We will contact authors of eligible studies on Alzheimer’s dementia and type 1 diabetes allocated to the control group to participate using four strategies, as per Dillman’s method [21] to optimize response rates and obtain IPD. First, authors will be sent an email requesting their IPD. Second, we will send four email reminders at 2-, 6-, 10-, and 14-week intervals after the initial email. Third, in week 7, we will send a reminder by post in addition to email. Fourth, in week 15 we will attempt to contact the corresponding author by phone. The duration of our study will be 19 weeks in total (see Fig. 2).

#### Intervention group

Using the same approach used for the control group, we will contact authors using the four approaches described above. Authors allocated to the intervention group will be additionally provided with a financial incentive (*CAD*100). Each participant allocated to the intervention group will receive an upfront *CAD*100 incentive as a gift certificate from Amazon (www.amazon.com) with the first email notification. In the same email we will clarify that at the end of the RCT, we will offer the same financial incentive to the authors allocated to the control group.

We will send two different letters by email, one for each group, simultaneously to the authors. The same letters will be sent to the authors’ mail addresses, which will have been printed in two different files, one for each group. In both letters, we will ask authors of the original studies to be included in the group authorship on the understanding that they provide feedback on results and take part in writing and reviewing the systematic review manuscript for the final publication, as is common practice in collaborative IPD reviews [32–35]. At the end of the RCT, we will send a debriefing letter to all the authors who participated to our study. All authors who will share their data with us will be appropriately cited and they will be acknowledged in our final manuscript if they wish.

### Outcomes

Our primary outcome will be the proportion of RCT authors included in our published systematic reviews who provide complete IPD. We will define complete IPD as information on population, interventions, outcomes and randomization as outlined below for the two reviews:

1. Population: the type 1 diabetes RCTs should include: age, sex, pregnancy, initial baseline glucose control (e.g., baseline glycosylated hemoglobin level), presence of comorbid conditions, previous history of hypoglycemia, other medications used for each participant, drop-outs along with reasons for drop-out, and number of participants, and Alzheimer’s dementia RCTs should include: age, sex, severity of the Alzheimer’s dementia, previous response to treatment for Alzheimer’s dementia, presence of behavioral disturbance, comorbid conditions (e.g., stroke), other medications used for each patient, drop-outs along with reasons for withdrawal, and number of participants
2. Interventions: including allocated treatment and dosage
3. Outcomes: including event and date of event for severe hypoglycemia in the type 1 diabetes review and serious adverse events in the Alzheimer’s dementia review, as well glycosylated hemoglobin and Mini-mental State Examination values and measurement dates for type 1 diabetes and Alzheimer’s dementia respectively
4. Date of randomization for each participant and overall method of randomization for all study participants

If any of the above items are not provided in the data we receive, but have been collected according to the RCT’s protocol, the study’s dataset will be considered partially complete. These items were chosen as the most vital data for IPD analyses based on input from clinicians on the relevant systematic review team.

Our secondary outcomes will be the time taken to obtain the dataset and the completeness of data. We will determine the duration between the information request and the authors’ provision of their dataset to estimate the time required to obtain data from authors. In case the authors send multiple datasets (e.g., first received dataset is incomplete, but after exchanging several emails the final received dataset is complete) over a period of time, we will consider the last date of correspondence to estimate the time required to obtain IPD. The completeness of the received dataset is crucial to investigators, as missing data might prevent inclusion of a RCT in the meta-analysis. An IPD meta-analysis may be biased if it is based only on a subset of trials [13]. If an RCT author provides us with the requested information, but some variables are missing (e.g., age, sex, pregnancy) because these were not collected during the RCT, then we will consider the dataset complete if this was reported in the study protocol. This is because the data are not missing due to selection bias. However, in case the requested information is not provided and the data have been collected in the RCT, the dataset will be considered incomplete. In such cases, we will not be able to control for these variables for the particular RCT in the analysis.

### Ethical approval and confidentiality

Ethical approval for this RCT was obtained from the Research Ethics Board (Dr. David Mazer, Dr. Philip Berger, and Dr. Brenda McDowell) of St. Michael’s Hospital (see Appendix).We are conducting the RCT to examine the impact of incentivizing authors versus usual contact strategies to obtain original IPD, and we feel that disclosing this early on in a consent letter will bias our results. Instead, we intend to send authors a debriefing letter after they share their data with us, letting them know that they were part of an RCT and that they can withdraw their data from our analysis, if they wish. The information generated during our study will be confidential and limited to the purposes, as described in this protocol. We will request authors to share anonymized IPD only, where each patient will be linked to a specific identifier.

### Power and sample size

A sample size of at least 116 participants in total (58 per group) for evaluation will provide 80 % power at the 5 % level of significance (two-sided) to detect an increase in response rates from 30 % in the control group to 55 % in the experimental group with 1:1 allocation. This is based on studies examining response rates of surveys. We anticipate a large response difference (i.e., absolute increase of 25 %) between the two groups as large and upfront incentives have been shown to be more effective than no, small or promised monetary incentives [36–39]. James et al. [36] compared survey response rates between US$25 prepaid and US$25 promised and found that upfront payment of a cash incentive was significantly more effective (odds ratio (OR) 2.88, 95 % confidence interval (CI) (1.70, 4.89)). Similarly, Pit et al. [38] conducted a pairwise meta-analysis comparing monetary incentives versus no incentives in survey responses and found that cash incentives were more effective at increasing response rates (OR = 1.87, 95 % CI (1.55, 2.26)). Our response rate estimate for the control arm (30 %) was based on previous empirical findings for retrieval of missing data in meta-analysis [40]. We expect to have adequate power for a 25 % response difference between the groups, as the current number of authors to contact is 138 (i.e., 30 authors to contact for the type 1 diabetes systematic review and 108 authors for the Alzheimer’s dementia systematic review). We have experience in contacting authors, as this is a regular process to ask for additional aggregated data on the eligible studies to enhance clarity in a meta-analysis, and on average we have a good response rate (over 60 %).

### Data collection, management and statistical analysis

Two team members (AAV, HA) will independently assess the data retrieved to ensure the datasets are complete as defined in the Outcomes section. The assessment process will be done blind to the allocation using a computer-generated random number list. Conflicts will be resolved by discussion or involvement of a third member (SES, ACT). All IPD will be held on a password-protected database on a secure server at St. Michael’s Hospital. Access to data and authors’ responses will be restricted to the research team and will not be shared with any third parties.

The analysis will be performed on an intention-to-treat basis and participants will be analyzed in the arms they were allocated to, regardless of whether they received the intervention or not. Results will be reported according to the CONSORT statement [3]. We will describe the two groups in terms of their baseline characteristics, including sex (in RCTs the investigators are focused on the sex of the patient (biological) and not gender) of authors contacted, sponsor information, study size, risk of bias, treatments compared, magnitude and statistical significance of the treatment effect (as presented in the published trial), year of publication, country in which the study was conducted (according to first author), and journal in which the study was published, irrespective of whether the authors provided their IPD. The funding source will be categorized as: (1) industry-sponsored trials (funded by or authored by an employee of a pharmaceutical or other commercial organization), (2) publicly-sponsored trials (governmental sources and non-profit organizations, including universities, hospitals, and foundations), and (3) non-sponsored trials (no funding source) [41]. We will also categorize studies according to their sample size as small (fewer than 50 patients per arm), moderate (50–150 patients per arm), and large (more than 150 patients per arm) [42]. Similar to our previous systematic review [26], we will appraise the risk of bias using the Cochrane Risk of Bias tool [43]. Two investigators (HA and AAV/ACT) will independently assess the risk of bias in each included study, and any disagreements will be resolved by discussion with a third investigator (SES). If risk of bias is unclear, we will ask the author for clarification. Risk of bias will be assessed considering direct investigation of the IPD as described in the Preferred Reporting Items for Systematic Reviews and Meta-Analyses (PRISMA) extension to IPD checklist [44]. For example, randomization might seem of low risk of bias in the text, but when assessing this using IPD it might show that there is an unbalanced treatment allocation to groups, and vice versa. We believe that a small study size and high risk of bias are factors that may impact on the IPD retrieval.

In this RCT, we will summarize categorical data in each group using frequencies and percentages and 95 % CIs, and we will use the chi-square test or Fisher’s exact test as appropriate to compare them. The continuous characteristics of each group will be presented using means and 95 % CIs if approximately normally distributed, and medians and interquartile ranges (IQRs) if non-normally distributed. We will compare means of the two groups using the *t* test, and medians of the intervention groups using the Wilcoxon-Mann-Whitney test. We will also use summary statistics, e.g., OR for dichotomous data, mean difference for symmetrical continuous data, and ratio of geometric means for skewed continuous data, along with the corresponding 95 % CI. We will use the Shapiro-Wilk test to determine normality for each variable.

The primary data analysis will compare responses with complete IPD between experimental and control groups using ORs and corresponding 95 % CIs. We will calculate the OR for response as soon as we have completed all different strategies to contact the eligible authors, as describe in the control and intervention groups section. For the secondary outcome (time needed to share the data), we will calculate the mean and 95 % CI for both control and experimental groups, and then we will compare them using mean difference and 95 % CI. We will also describe the completeness of each study’s dataset using percentages and 95 % CIs, and compare the average completeness of IPD between groups using mean difference and 95 % CI. If authors are lost to follow-up (e.g., they promise they will share the data and we never hear back from them), a sensitivity analysis will be undertaken using a complete case analysis to assess the robustness of the findings with respect to missing data for all outcomes.

To examine whether different study characteristics influence the probability of providing complete IPD as specified in the primary outcome, we will use binary logistic regression adjusting for any confounding variables (e.g., small study size, high risk of bias, funding) as appropriate. We will start with bivariate regression analyses (including one dependent and one independent variable) and then for significant moderators we will simultaneously enter them into multiple regression models as long as the minimum number of cases per independent variable is 10. The significance of the variables in the model will be evaluated with the Wald chi-square test and determination of ORs with the associated 95 % CIs. Similarly, to investigate the potential influence of the aforementioned confounding variables on the secondary outcome time needed to share the data, we will apply a Cox regression model adjusting for these explanatory variables. Statistical significance will be assessed at the 0.05 level (two-tailed). All statistical analyses will be conducted by the lead author (AAV).

## Discussion

To date, there has been a steady increase in publication of systematic reviews that conduct an IPD meta-analysis [13], but obtaining IPD is time-consuming and costly, and depends on the authors’ willingness to share their data. Reviewers who wish to complete an IPD meta-analysis are often not able to obtain the IPD of all studies, and hence results might be prone to several biases [14, 45, 46]. To the best of our knowledge, this is the first experimental study that explores the effects of a cash incentive to encourage authors of eligible studies to submit their IPD. Results of this study will establish feasibility and provide evidence on the level of response with respect to different study characteristics. In addition, we will capture the most optimal and practical strategies for maximizing the amount of IPD obtained. Previous research evaluating the influence of financial incentives on survey requests showed that such incentives improve response rates considerably (average improvement between 15 and 19 %) [21–24]. This is particularly important, as the study’s sample may not be random according to the patient population, which may result in biased study results and conclusions. A limitation of this study is that we used survey research to inform our sample size, as to the best of our knowledge there are no studies assessing response rate in retrieving IPD from collaborative studies using financial incentives.

Findings of our study will contribute to the future planning of IPD meta-analyses given that the IPD retrieval is part of their research process. We will be able to provide guidance on ways that IPD might be obtained. This research will help customize approaches to planning and conducting IPD meta-analyses, including estimation of the time needed and effective ways to collect the IPD. This will help reviewers to effectively plan their timelines, which may increase the use of IPD in meta-analysis and network meta-analysis. We will disseminate the results of this study in an open access scientific journal and present our results at conferences.

### Trial status

As of October 2015, we have updated the literature searches, and finalized the screening process for our systematic reviews, and randomized the authors of the eligible studies. We plan to start contacting the authors in the beginning of February 2016.

## Appendix

Ethical approval obtained from the Research Ethics Board to conduct this randomized controlled trial.

## Abbreviations

CI: confidence interval
CONSORT: Consolidated Standards of Reporting Trials
IQR: interquartile range
IPD: individual patient data
PRISMA: Preferred Reporting Items for Systematic Reviews and Meta-Analyses
RCT: randomized controlled trial
SPIRIT: Standard protocol Items: Recommendations for Interventional Trials.
